# Diagnostic validation of a rapid and field-applicable PCR-lateral flow test system for point-of-care detection of cyprinid herpesvirus 3 (CyHV-3)

**DOI:** 10.1371/journal.pone.0241420

**Published:** 2020-10-30

**Authors:** Finn N. Loose, André Breitbach, Ivo Bertalan, Dana Rüster, Uwe Truyen, Stephanie Speck

**Affiliations:** 1 Frankenförder Forschungsgesellschaft mbH (FFG), Berlin, Germany; 2 Institute of Animal Hygiene and Veterinary Public Health, University of Leipzig, Leipzig, Germany; 3 Institute of Plant Physiology, Martin-Luther-University Halle-Wittenberg, Halle, Germany; 4 Milenia Biotec GmbH, Gießen, Germany; Xiamen University, CHINA

## Abstract

Koi herpesvirus disease (KHVD) is a highly infectious disease leading to outbreaks and mass mortality in captive and free-ranging common carp and koi carp. Outbreaks may result in high morbidity and mortality which can have a severe economic impact along the supply chain. Currently, control and prevention of KHVD relies on avoiding exposure to the virus based on efficient hygiene and biosecurity measures. An early diagnosis of the disease is crucial to prevent its spread and to minimize economic losses. Therefore, an easy-to-handle, sensitive, specific and reliable test prototype for a point-of-care detection of KHV was developed and evaluated in this study. We used a multiplex-endpoint-PCR followed by a specific probe hybridization step. PCR-products/hybridization-products were visualized with a simple and universal lateral flow immunoassay (PCR-LFA). Fifty-four gill tissue samples (KHV-positive n = 33, KHV-negative n = 21) and 46 kidney samples (KHV-positive n = 24, KHV-negative n = 22) were used to determine diagnostic sensitivity and specificity of the PCR-LFA. In addition, the usability of PCR-LFA to detect CyHV-3-DNA in gill swabs taken from 20 perished common carp during a KHVD-outbreak in a commercial carp stock was examined. This assay gave test results within approximately 60 min. It revealed a detection limit of 9 KHV gene copies/μl (95% probability), a diagnostic specificity of 100%, and diagnostic sensitivity of 94.81% if samples were tested in a single test run only. PCR inhibition was noticed when examining gill swab samples without preceding extraction of DNA or sample dilution. Test sensitivity coud be enhanced by examining samples in five replicates. Overall, our PCR-LFA proved to be a specific, easy-to-use and time-saving point-of-care-compatible test for the detection of KHV-DNA. Regarding gill swab samples, further test series using a higher number of clinical samples should be analyzed to confirm the number of replicates and the sample processing necessary to reveal a 100% diagnostic sensitivity.

## Introduction

In the past few decades, aquaculture has evolved as an important business segment worldwide. No global food production sector is growing faster. In 2016, over 80 million tons of food fish have been produced with a value of 231.6 billion US Dollar [1]. Among the finfish, the common carp (*Cyprinus carpio carpio*) accounted for the third most produced species worldwide [1]. Koi carp (*Cyprinus carpio* var. *koi*) the ornamental variant of common carp has a high breeding and economic value and belongs to the most popular ornamental pond fish worldwide [2].

Koi herpesvirus disease (KHVD) is a highly infectious disease leading to outbreaks and mass death in captive and free-ranging common carp and koi carp [3, 4]. The causative agent is Cyprinid herpesvirus 3 (CyHV-3), also known as koi herpesvirus (KHV), genus *Cyprinivirus*, *Alloherpesviridae* [5]. First descriptions of the disease originated from Israel, the USA, and Germany [6, 7]. Clinical signs include gill necrosis, discolored patches on the skin, erratic swimming and enophthalmos [8]. Water temperatures between 22°C and 26°C favor KHVD outbreaks [9]. CyHV-3 causes latent infection which can be reactivated someday after the initial exposure. Healthy but latently infected carrier-fish constitute a considerable risk for the spread of CyHV-3 during trade and fish farming procedures [9–13]. KHVD outbreaks may result in high morbidity and mortality which can have a severe economic impact along the supply chain [9]. Because of its epizootic appearance, KHVD is listed as a notifiable disease in Germany [13, 14] and by the World Organization for Animal Health (OIE) (https://www.oie.int/en/animal-health-in-the-world/oie-listed-diseases-2019/) [15].

A safe and effective vaccine is currently not widely available. Hence, control and prevention of KHVD relies on avoiding exposure to the virus based on efficient hygiene and biosecurity measures [16]. An early diagnosis of the disease is crucial to prevent its spread and minimize economic losses. There are several methods for targeted surveillance and diagnosis of KHV but PCR is the recommended technique for reasons of availability, ease-of-use, diagnostic specificity and sensitivity [16]. A rapid and reliable pond-side test would shorten the time required for routine diagnostic. Lateral flow immunoassays (LFA) are widely used in human and veterinary medical diagnostic investigations [17] and can be used for primary screening at the point of care (POC) need.

The aim of this study was to develop and evaluate an easy-to-handle, sensitive, specific and reliable test prototype for a POC detection of KHV. The overall assay performance should be comparable to approved reference methods. Therefore, we chose a combination of DNA-amplification via multiplex-endpoint-PCR followed by a specific probe hybridization step, then visualized with a simple and universal lateral flow immunoassay (PCR-LFA).

## Material and methods

### PCR-lateral flow analysis procedure (PCR-LFA)

Polymerase chain reaction (PCR) was designed according to Gilad et al. (2004) [18] with some modifications (Table 1). The PCR-LFA was divided into three steps: DNA-amplification using multiplex-endpoint-PCR, post-PCR hybridization, and LFA.

**Table 1:** Oligonucleotides used in this study.

Name	Sequence 5’ → 3’	Modification	Reference
KHV 86F	GACGCCGGAGACCTTGTG	none	[18]
KHV 163R FITC	CGGGTTCTTATTTTTGTCCTTGTT	5’ FITC	[18]
KHV 109P (rc) BIO	CTTCCTCTGCTCGGCGAGCACG	5’ Biotin	[18]*
KHV-Helper 1	GACGCCGGAGACCTTGTG	none	this work
KHV-Helper 2	GCAAAAAGAACAAGGACAAAAATAAGAACCC	none	this work

Amplification of KHV-specific PCR fragments (78 bp) was performed using primers KHV 86F und KHV 163R FITC. After PCR, the hybridization solution H was added to the PCR-products. Solution H included the biotinylated KHV probe (KHV 109P (rc) BIO) and the hybridization helper 2.

*The biotinylated probe was reversed in comparison to [18].

**Fig 1:**
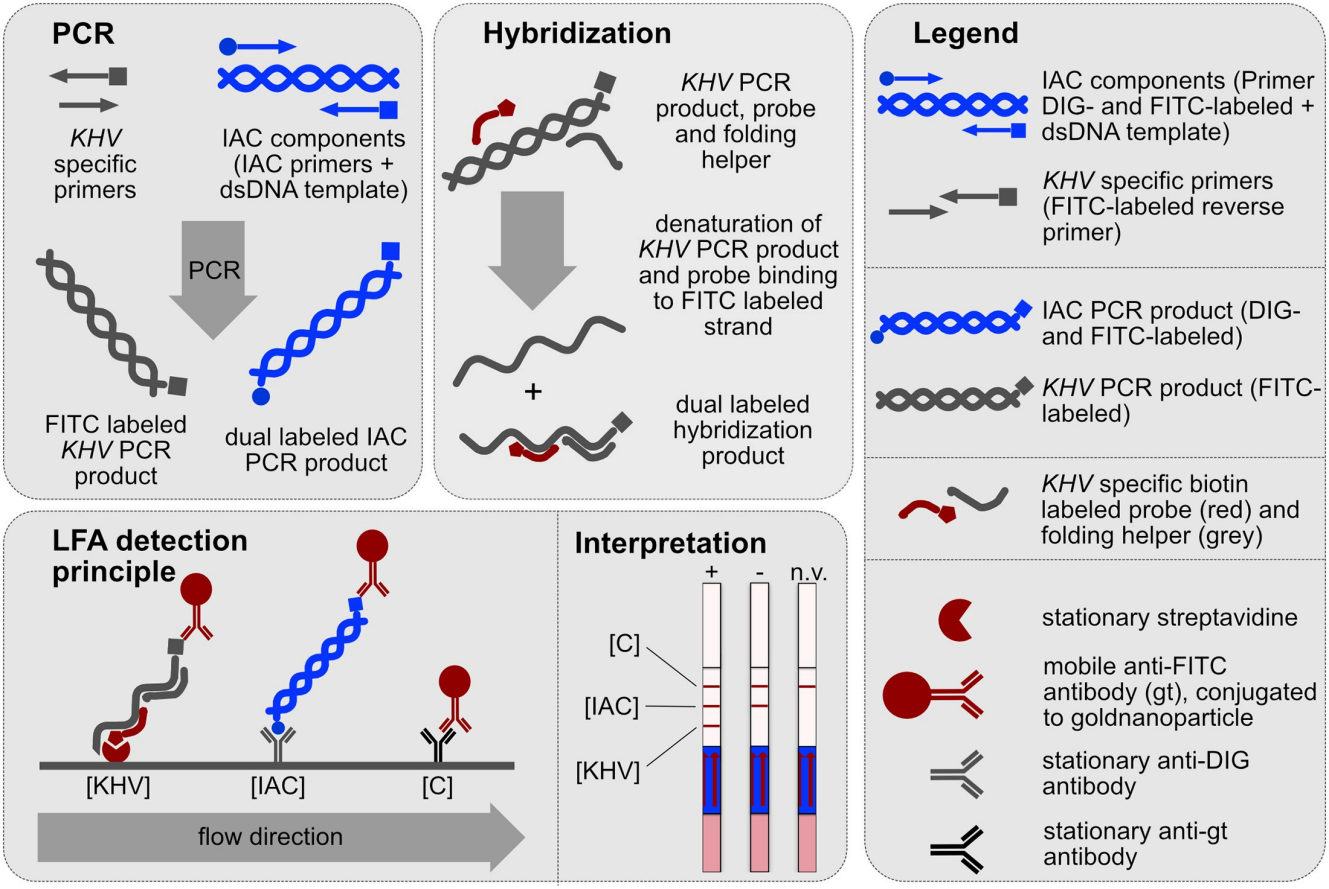
Detection principle of the PCR-LFA. **PCR:** A KHV-specific FITC-labeled PCR product is amplified from KHV-DNA. The amplified internal amplification control [IAC] PCR product is double-labeled (DIG and FITC). **Hybridization:** During post-PCR hybridization, the biotin labeled probe binds to the FITC-labeled strand of the KHV-specific PCR product. This results in a hybridization product which is detectable via LFA. **LFA detection principle:** The KHV-specific hybridization product is immobilized due to biotin-streptavidin interaction. The IAC PCR product is bound by a stationary anti-digoxigenin [DIG] antibody. The mobile gold-conjugate contains anti-FITC antibodies which specifically bind to the FITC-labeled immobilized PCR products on the pad. Accumulation of gold nanoparticles leads to the appearance of visible test lines. The immunoassay control line [C] is formed due to binding of the mobile anti-FITC antibodies (from goat; gt) by a stationary anti-goat antibody. Appearance of the control line confirms general functionality of the LFA. **LFA Interpretation:** [+] The test is interpreted as positive if the KHV-specific test line appears. [–] A negative result is defined by absence of the KHV-test line while the IAC-signal is present. [n.v.] If the KHV- and the IAC-signal are absent, the test is interpreted as not valid.

For PCR-LFA, three different ready-to-use solutions (A, B and H) were developed. Solution A (Milenia Biotec GmbH) consisted of two KHV-specific primers (KHV 86 F, KHV 163R FITC) and a “carp-independent” internal amplification control (IAC). The latter was composed of digoxigenin (DIG)- and fluorescein isothiocyanate (FITC)-labeled primers and the IAC template (Milenia Biotec GmbH, Gießen, Germany). It was highly compatible to the KHV-PCR of Gilas et al. (2004) [18] without decreasing assay sensitivity (data not shown). Solution B (Milenia Biotec GmbH) contained 2x polymerase-mix with hot-start DNA-polymerase, dNTP’s, and PCR-buffer. Solution H was used for post-PCR hybridization. It consisted of 2.9x saline-sodium citrate (SSC) buffer, 18.2 nM biotinylated KHV probe (KHV 109P (rc) BIO), and 181.8 nM KHV-Helper 2. PCR was carried out in a 25 μl-mixture of Solution A (7.5 μl), Solution B (12.5 μl), and 5 μl template in a Sensoquest Labcycler 48s (Sensoquest GmbH, Göttingen, Germany) under the following conditions: initial denaturation for 240 seconds at 95°C followed by 40 cycles of 15 seconds at 95°C, 20 seconds at 60°C, 10 seconds at 72°C. The PCR protocol was stopped at 8°C after a final elongation of 10 seconds at 72°C. Subsequently, 55 μl of Solution H were added to each PCR-tube, gently mixed, and the hybridization step was started (60 seconds of denaturation and 5 min of incubation at 50°C). Thereafter, a LFA-dipstick (HybriDetect 2T, Milenia Biotec GmbH) was placed in each PCR-tube whilst maintaining cycler temperature at 50°C for 10 min. Dipsticks were removed after 10 min of incubation and results documented photographically. Results were evaluated manually by naked eye. The HybriDetect 2T dipstick is a universal lateral flow dipstick for simultaneous detection of two different analytes. Each dipstick comprised three lines: immunoassay control line (C-line), IAC-test line, and KHV-test line. Appearance of the C-line indicated functionality of the LFA. Appropriate DNA amplification was confirmed by the IAC-test line. This feature is crucial for discrimination between a valid negative result and an inhibited PCR. The KHV-test line indicated the presence of a KHV-specific PCR product. The detection principle of the PCR-LFA is given in Fig 1.

### Viruses

KHV isolates ‘Israel’ (HP 951) and ‘Taiwan 832’ were grown in common carp brain cells (CCLV-RIE 816) as described elsewhere [19, 20]. Spring viremia of carp virus (SVCV; isolate RC 56/70, RVB-405), pike fry sprivivirus (PFRV; isolate PFR42, RVB-0431), and viral hemorrhagic septicemia virus (VHSV; isolate Fi13, RVB-0417) were grown in fat head minnow caudal trunk cells (FMH; CCLV-RIE 57) maintained in Minimal Essential Medium Hanks’ Balanced Salts (MEM HBSS; Life Technologies GmbH, Darmstadt, Germany) adjusted to 850 mg/l NaHCO_3_ (Life Technologies GmbH), 10% fetal bovine serum (FBS; Sigma Aldrich Chemie GmbH, Schnelldorf, Germany) without CO_2_ at 20°C. Fat head minnow cells (EPC; CCLV-RIE 173) grown in a 1:1-mixture of MEM HBSS and MEM Earle’s BSS (Life Technologies GmbH) containing 1.5 g/l NaHCO_3_, 1x non-essential amino acids (Life Technologies GmbH), 120 mg/l sodium pyruvate (Life Technologies GmbH), and 10% FBS (Sigma Aldrich Chemie GmbH) were used to culture infectious haematopoietic necrosis virus (IHNV; isolate DF 11/95, RVB-0086) at 20°C without CO_2_. Cells and viruses were obtained from the Collection of Cell Lines in Veterinary Medicine, Friedrich-Löffler-Institute, Greifswald-Insel Riems, Germany. Carp edema virus (CEV)-positive gill samples were kindly provided by Dr. A. Muluneh, LUA Sachsen, Dresden, Germany. Nucleic acids were prepared using the QIAamp DNA Mini Kit and the QIAamp Viral RNA Mini Kit (Qiagen, Hilden, Germany) according the manufacturer’s instructions. DNA of CyHV-1 and CyHV-2 was kindly provided by Dr. M. Adamek, Fish Disease Research Unit, Centre for Infection Medicine, University of Veterinary Medicine in Hanover, Hanover, Germany.

### Carp tissue

Gill and kidney samples were obtained from KHV-negative and experimentally KHV-infected common carp (K2). All carp were part of another study which was approved by the Animal Welfare Officer of the Veterinary Faculty at Leipzig University and it was performed with permission of the Landesdirektion Sachsen (TVV A09/13, TVV A09/14). Experimental infection was carried out using KHV isolate ‘Israel’. The study was published in 2016 elsewhere [19].

### Analytical sensitivity of the KHV PCR-LFA

To determine the limit of detection (LOD), the following concentrations of a plasmid standard (cTL-21) harboring a 484 bp-fragment of the KHV genome [20] were tested in five replicates each: 606 copies/μl, 60.6 copies/μl, 30.3 copies/μl, 6.06 copies/μl, 4.55 copies/μl, 3.03 copies/μl, 1.52 copies/μl, 0.6 copies/μl, and 0.06 copies/μl. A regression analysis with the probit model was imposed using the statistics software R version 3.0.2 [21]. The ≥95% LOD was calculated using the dose.p function of the MASS package [22]. Results were compared to the reference PCR (quantitative real-time PCR; qPCR) according to Gilad et al. (2004) [18] modified by Gaede et al. (2017) [20]. The LOD of the qPCR using the cTL-21 plasmid was 10.61 copies per 5 μl [20].

### Analytical specificity of the KHV PCR-LFA

Analytical specificity was tested using CyHV-1, CyHV-2, and CEV. In addition, viruses (SVCV, IHNV, VHSV, PFRV), bacteria (*Staphylococcus aureus*, *Bacillus cereus*, *Aeromonas hydrophila*, *Francisella* (*F*.) *noatunensis* subsp. *orientalis*, *Pseudomonas koreensis*) and an alga (*Chlamydomonas reinhardtii*, wild type strain CC-124) known to cause disease in carp or associated with the aquatic environment were tested. Moreover, DNA prepared from food fish (salmon, rainbow trout, redfish) was tested. DNA from *F*. *noatunensis* subsp. *orientalis* was kindly provided by the Bundeswehr Institute of Microbiology, Munich, Germany.

### Repeatability (intra-assay variance)

To test for precision and robustness [23] of the PCR-LFA, DNA extracted from gills (n = 3) and kidneys (n = 3) was analyzed in triplicates in the same assay at one day. According to the reference qPCR [18, 20] these samples were categorized as KHV-negative (C_t_-value 0), KHV-weakly positive (C_t_-value >36), and KHV-distinct positive (C_t_-value <24) (S1 Table).

### Reproducibility (inter-assay variance)

In order to test for variation in results between runs [23] the samples used for intra-assay variance were tested as single-preparations on two consecutive days.

### Diagnostic specificity and sensitivity

Fifty-four gill tissue samples (KHV-positive n = 33, KHV-negative n = 21) and 46 kidney samples (KHV-positive n = 24, KHV-negative n = 22) were used to determine diagnostic sensitivity and specificity of the PCR-LFA (S1 Table). Samples were tested previously with the qPCR using the protocol published by Gaede et al. (2017) [20]. Briefly, the qPCR was performed in a Stratagene MX3000P with the following cycling condition: initial denaturation at 95°C, 15 min; followed by 40 cycles of denaturation at 95°C for 20 s; annealing at 60°C for 30 s and final elongation at 72°C for 30 s. The total reaction volume (25 μl) contained 2x QuantiTect Multiplex NoROX Mastermix (Qiagen), 0.4 μM of KHV-86f and KHV-163r and 0.2 μM of KHV-109 probe. Five μl of DNA were used as template for qPCR amplification. Each sample was tested once by PCR-LFA. If results differed from qPCR, samples were tested in three and five replicates.

In addition, the usability of the PCR-LFA for the detection of CyHV-3-DNA in gill swabs taken from 20 deceased common carp during a KHVD-outbreak in a commercial carp stock was examined. Dry cotton swabs were used for sampling. Swabs (S1 Table) were transported cooled (+4°C) to the laboratory and PCR-LFA was performed at the same day. Each swab was placed in 250 μl sterile phosphate-buffered saline (pH 7.4) for 10 min, rinsed, and the eluate was submitted to PCR-LFA without further processing.

All PCR-LFA results were compared to the gold standard reference qPCR [18, 20]. Specificity, sensitivity, positive predictive value (probability that a test-positive fish is actually positive), and negative predictive value (probability that a test-negative fish is a true-negative) of the PCR-LFA were calculated according to Thrusfield (2018) [24].

## Results

### PCR-LFA development

#### PCR adaption and cross primer-dimer analysis

**Fig 2:**
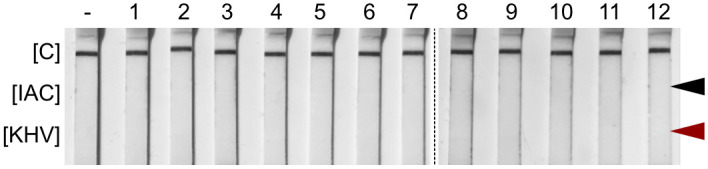
Cross primer-dimer analysis. Test for nonspecific interaction between labeled primers or probes resulting in PCR-independent signals in LFA. The following reaction components were tested: Lane [–] negative control—without primers or probes; lane [1] KHV 163R FITC; lane [2] KHV 109P (rc) BIO; lane [3] IAC-Mix (1x); lane [4] KHV 163R FITC + KHV 109P (rc) BIO; lane [5] KHV 163R FITC + IAC-Mix (1x); lane [6] KHV 109P (rc) BIO + IAC-Mix (1x); lane [7] KHV 163R FITC + KHV 109P (rc) BIO + IAC-Mix (1x); lanes [8–12] 2x KHV-primer mix + KHV 109P (rc) BIO. The red arrow indicates the location where the KHV-specific test line [KHV] would appear. The black arrow indicates the location where the internal amplification control line [IAC] would be seen. Fig 2 was prepared from two raw images, i.e. S3 and S4 Figs.

In order to prevent any nonspecific interaction between primers and probes, every labeled component of the detection system was tested. Approximately seven pmol of each labeled oligonucleotide were co-incubated under hybridization conditions and analyzed by LFA. No cross primer or probe interaction was observed for all the combinations tested (Fig 2). This confirmed compatibility of the primers and probes developed by Gilad et al. (2004) [18] with the PCR-LFA test principle.

#### Optimization of hybridization conditions

**Fig 3:**
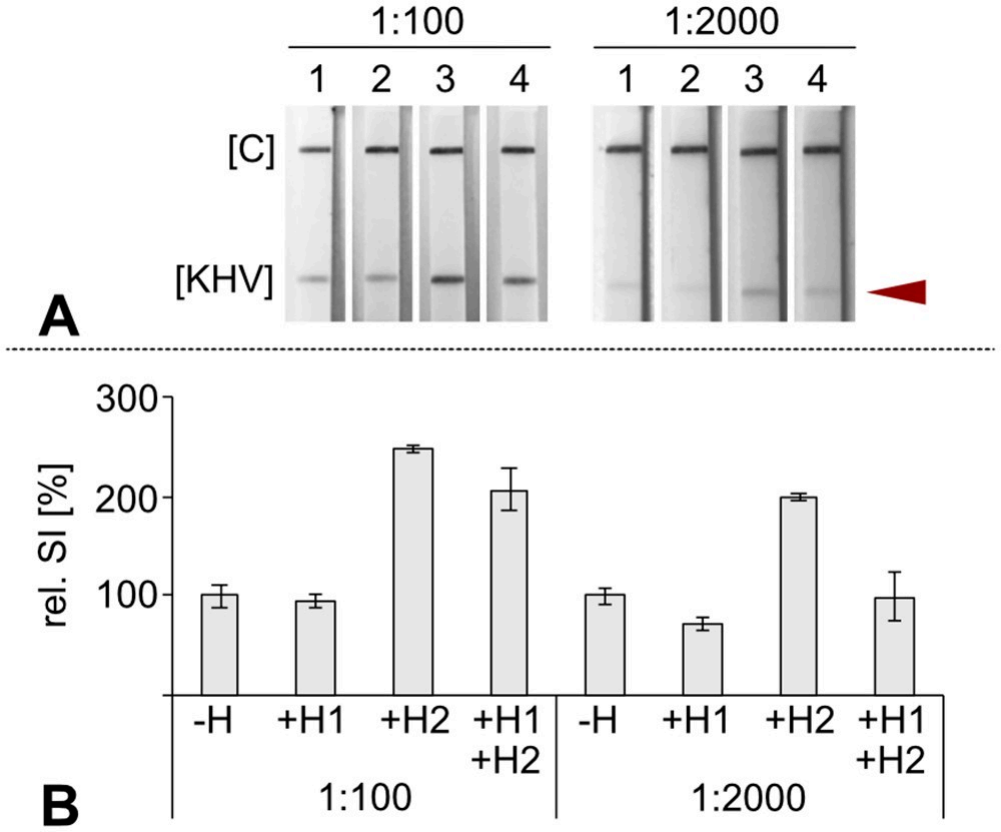
Influence of unlabeled helper oligonucleotides on hybridization efficiency. Two helper oligonucleotides, KHV-Helper 1 (H1) and KHV-Helper 2 (H2), were tested under hybridization conditions. Ten pmol of each helper was added to each hybridization mix including two pmol of the detection probe KHV 109P (rc) BIO. **(A)** Each helper combination was tested with two KHV-PCR product concentrations (dilution 1:100 and 1:2,000, respectively) in triplicate. Representative test strips are shown: lane 1—without helpers (-H); lane 2—with H1 (+H1); lane 3—with H2 (+H2); lane 4—with H1 and H2 (+H1+H2). [C] immunoassay control line; [KHV] KHV-specific test line indicated by a red arrow. **(B)** Signal intensities are shown as relative intensities (rel. SI [%]). The intensity of the test line on strip 1 (Fig 3A, lane 1) without helpers was treated as reference. Mean value was set to 100%. The raw images used to prepare Fig 3 can be found as S5 and S6 Figs.

Physicochemical optimization of hybridization parameters was carried out, i.e. adjustment of ionic strength, time for denaturation, hybridization temperature and duration of the hybridization reaction (data not shown). The addition of helper oligonucleotides to the hybridization solution considerably increased KHV-specific signal intensity on the dipstick (Fig 3). These helpers were designed to match the FITC labeled DNA-strand in regions adjacent to the probe binding site to minimize potential secondary structures of the 78 bp target strand. The usage of the KHV-Helper 2, which was complementary to the 5’ end of the FITC labeled strand, led to at least 200% more intense KHV-specific signals on the test strip. The effect of unlabeled helpers is not a new phenomenon and has been described for rRNA based hybridization techniques elsewhere [25–27].

Optimal hybridization conditions were defined as follows: 55 μl of solution H (2.9x SSC-solution containing 10 pmol helper and 1 pmol KHV 109P(rc) Bio per hybridization reaction) were added to 25 μl of PCR amplicons (i.e. final SSC concentration 2x). Denaturation was carried out at 50°C for 1 min followed by 5 min of hybridization at 50°C in the PCR cycler. Thereafter, dipsticks were placed into the PCR tubes and LFA was carried out in the thermal cycler while constantly heating the mix at 50°C. Representative dipsticks showing signal intensity during optimization can be seen in Fig 3A. Signal intensities were additionally quantified via ImageJ (https://imagej.nih.gov/ij/) and results are displayed in Fig 3B. Comprehensive results are given in S1 Fig.

#### Development of a PCR-LFA protocol

**Fig 4:**
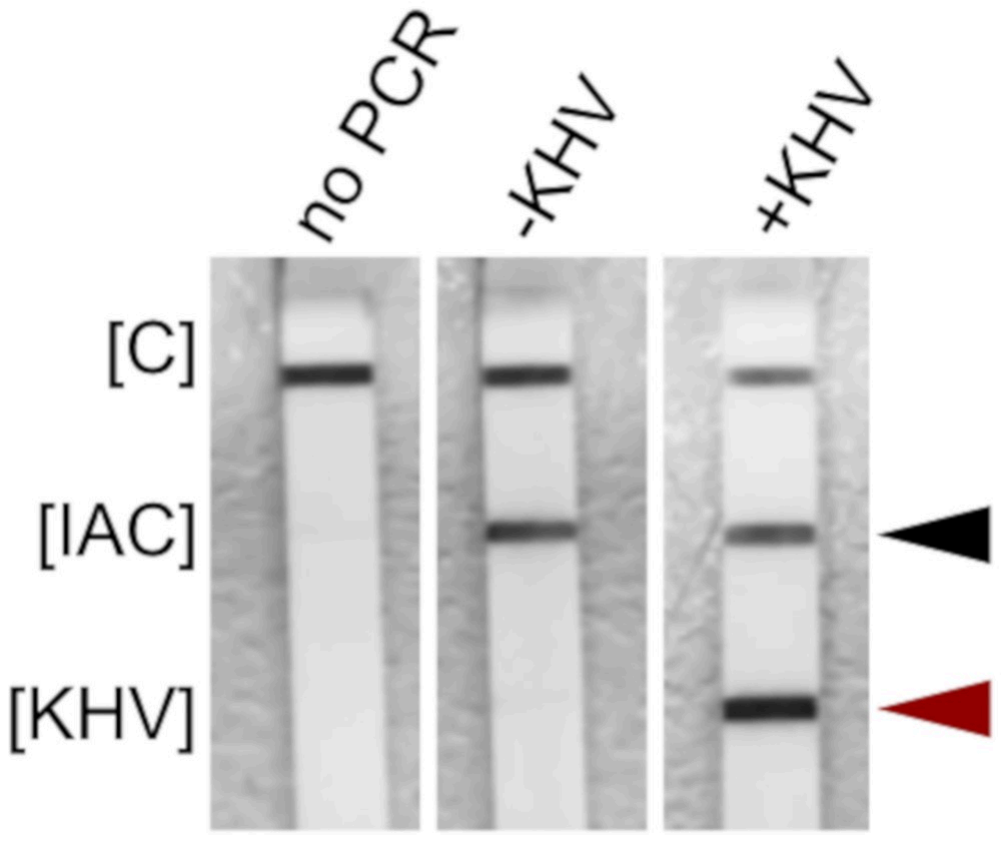
Functionality of KHV PCR-LFA prototype. No PCR—no PCR control; -KHV—no template control; +KHV—positive control (approx. 1x10^5^ KHV copies/μl); [C]—immunoassay control; [IAC]—internal amplification control line (black arrow); [KHV]–KHV-specific test line (red triangle). Comprehensive results are given in S2 Fig and are based on the raw image file S7 Fig.

A well working PCR-LFA protocol was developed for screening at the POC need. The protocol was based on the HybriDetect 2T (Milenia Biotech GmbH), which is a ready-to-use, simple and universal lateral flow device to develop quick test methods. Its test principle is based on lateral flow technology using gold particles. The developed reagents were ready-to-use and therefore easy to handle which constitutes a prerequisite for POC analyses. Hereby, the test became less laborious and less prone to handling errors. The simple 3-step PCR protocol ensures assay transfer to various benchtop or portable PCR machines. The PCR-LFA protocol allowed a sensitive detection of KHV-DNA in approximately 1 h analysis time with a hands-on time less than 10 min (without sample preparation). Preliminary experiments showed a satisfying performance in terms of sensitivity and specificity. The overall functionality of the KHV PCR-LFA protocol is presented in Fig 4.

### PCR-LFA validation

#### Analytical sensitivity of PCR-LFA

**Fig 5:**
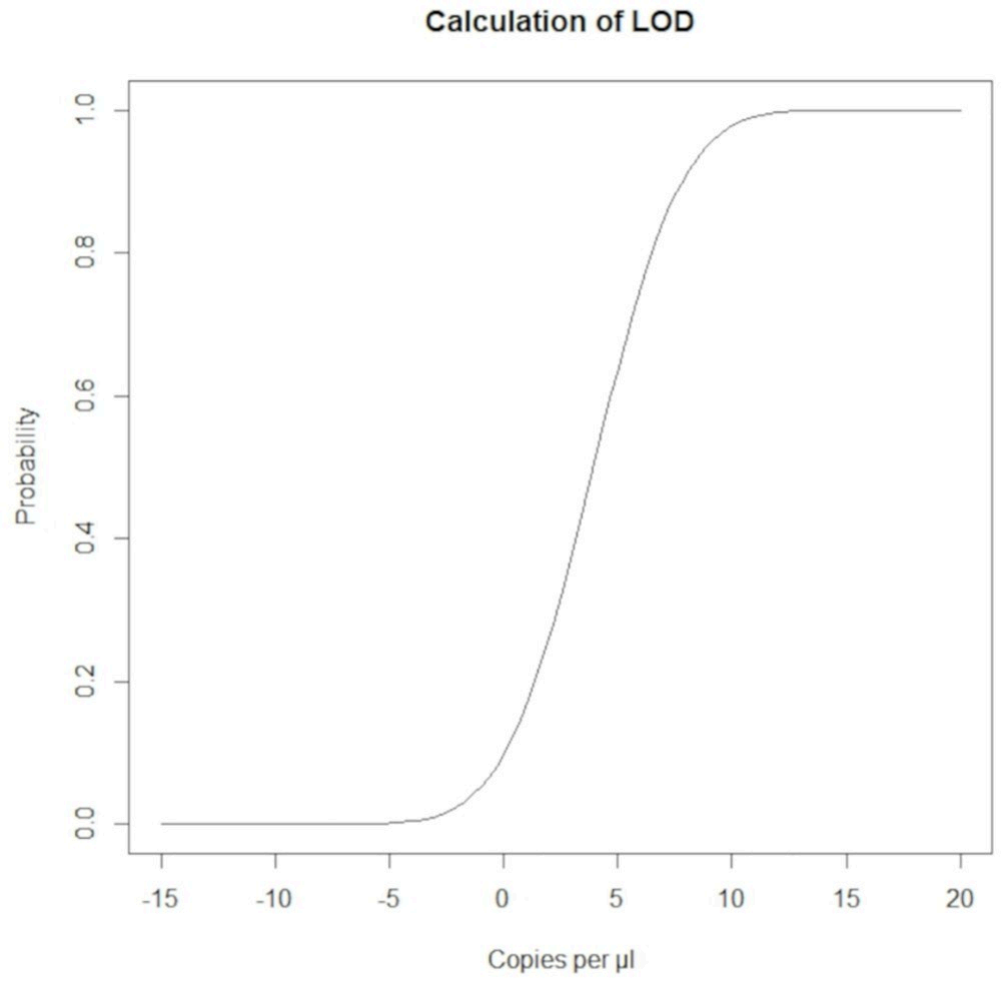
Graphical presentation of the limit of detection determined using serial dilutions of the cTL-21 plasmid standard.

The concentrations of the plasmid standard cTL-21 and the results obtained for analytical sensitivity are given in Table 2. Based on the number of positive tests per total number of tests (i.e. n = 5) the LOD was calculated. The LOD (95% probability) determined for the PCR-LFA was 9 copies/μl (Fig 5), i.e. 45 copies/5 μl.

**Table 2:** Concentrations of the plasmid standard cTL-21 tested in five replicates using PCR-LFA.

Gene copies/μl	n tests positive/total number of tests (n = 5)
606	5/5
60.6	5/5
30.3	5/5
6.06	4/5
4.55	1/5
3.03	4/5
1.52	2/5
0.6	0/5
0.06	0/5

#### Analytical specificity of PCR-LFA

**Fig 6:**
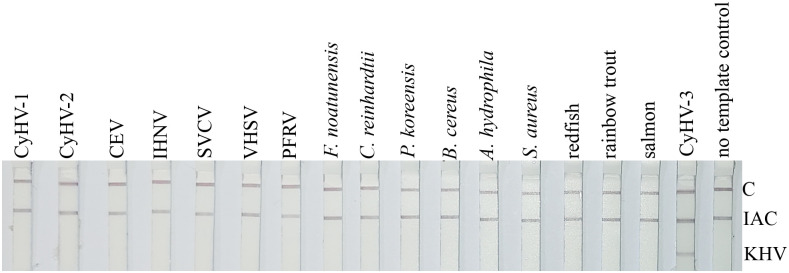
Analytical specificity of the KHV PCR-LFA. None of the samples from viruses, bacteria, food fish, and the alga gave a KHV-specific signal. KHV—indicates the location of the KHV-specific test line. IAC—indicates the location of the internal amplification control line. General functionality of the LFA is confirmed by the appearance of the immunoassay control line—C. The raw image file is given as S8 Fig.

No false positive signals were observed with any of the virus-cell culture samples, viral, bacterial, and fish DNA or the alga (Fig 6). Hence, test strips only show the IAC-line and the C-line indicating a valid test performance. This was also seen for the no template control. In addition to these lines, the KHV-specific test line was present in the KHV positive-control.

#### Intra- and inter-assay variability

There was no variation seen in PCR-LFA results obtained for positive and negative samples within and between runs. All KHV-positive and -negative samples revealed either positive or negative results when tested in triplicate within the same PCR-LFA run (intra-assay variability) as well as when tested in PCR-LFAs at two consecutive days (inter-assay variability). Results are given in Table 3.

**Table 3:** PCR-LFA results obtained for intra- and inter-assay variability.

Sample	ID	qPCR C_t_-value	KVH copies/μl	Intra-assay variability	Inter-assay variability
				n positive/n replicates	result on day 1/day 2
gill	IA-8	18.33	7.22x10^6^	3/3	positive/positive
gill	UI-44	36.56	4.56x10^1^	3/3	positive/positive
gill	D-22	0	0	0/3	negative/negative
kidney	IA-8	23.86	1.92x10^5^	3/3	positive/positive
kidney	IB-44	36.45	5.02x10^1^	3/3	positive/positive
kidney	D-22	0	0	0/3	negative/negative

#### Diagnostic sensitivity and specificity

Diagnostic sensitivity and specificity were tested using DNA from a total of 100 tissue samples from common carp with known disease status: i.e. 43 KHV-negative samples from KHV-negative carp and 57 KHV-positive samples from experimentally CyHV-3-infected carp. In addition, 20 gill swab samples from deceased common carp of a confirmed KHVD outbreak were tested. All samples were examined in a single test run. PCR-LFA accurately detected no KHV-DNA in samples from KHV-negative carp assuming a diagnostic specificity of 100%. Of 77 KHV-positive samples, 55/57 tissue specimen and 18/20 gill swab eluates were precisely identified as positive (diagnostic sensitivity = 94.81%). The two false-negative tissue samples (gill UI-55, kidney UI-55; S2 Table) were examined again each in three replicates. The gill sample was then determined as positive in 1 out of 3 replicates whereas the kidney sample remained negative in all three replicates. When tested in five replicates, the latter reacted positive in 1 out of 5 replicates. Of the two gill swabs (no. 10 and no. 11) negative by PCR-LFA, swab eluate no. 10 remained negative after repeated testing. The eluate of swab no. 11 completely inhibited the multiplex-endpoint-PCR which became obvious by a missing IAC-line. The IAC was also affected by another five swab eluates but the PCR-LFA revealed a KHV-positive result. When eluates were tested at a dilution of 1:10 or using extracted DNA, inhibition disappeared and the 20 samples were accurately identified as positive. The positive and negative predictive values for the single test run were 100% and 93.5%, respectively.

## Discussion

Sensitive and rapid POC diagnostic tests are of crucial importance for managing and mitigating the spread of infectious diseases. The latter is of paramount importance for global food security [28]. Since its first description in the late 1990s KHVD rapidly spread worldwide. Diagnosis of KHVD in clinically affected fish can currently be achieved by several methods, but some of them are not reliable (e.g. virus isolation in cell culture) or not validated (e.g. immunodiagnostic methods) [16]. Most commonly, PCR-based assays specific for KHV are used for detection of KHV in fish tissues [16]. They require sophisticated equipment and it will take a few days until results are available. There is a rapid (15 min) pondside test available for the detection of KHV-antigen in swab samples from gill tissues (FASTest^®^ KOI HV, Megacor Diagnostik GmbH, Hörbranz, Austria) which is based on an immunochromatographic “sandwich principle”. Its specificity and sensitivity are given as 100% and 71.7% (compared to the PCR according to Bercovier et al., 2005 [29]), respectively. The detection limit is given as 10^4^−10^6^ KHV/ml (Megacor Diagnostik GmbH). The PCR-LFA used in the study at hand was less rapid (approximately 60 min) but with identical specificity, a much lower detection limit (i.e. 9 gene copies/μl), and a higher analytical sensitivity. Although the reference qPCR [20] had a much higher sensitivity (2 copies/μl), most of the KHV-positive samples were also correctly identified by PCR-LFA. When used at pondsite, gill swab samples would be the most appropriate specimen. Our results show that these samples might inhibit the PCR amplification process. This was prevented by diluting the sample at 1:10. Therefore, a dilution at 1:10 might be recommended when testing gill swab samples. The validation of the PCR-LFA further revealed that a single test might result in false-negative findings. With our test-panel, diagnostic sensitivity was increased by testing five replicates per sample. This highlights the necessity for further test series at POC conditions.

Our results were similar to other studies using lateral flow assay strips for the rapid detection of KHV [30, 31]. The latter based their diagnostic systems on the loop mediated isothermal amplification and the isothermal DNA amplification assay using recombinase polymerase amplification [30, 31]. In contrast, we based our assay on a more common amplification technique, the PCR.

Generally, PCR is defined as the first and most accurately described *in vitro* DNA amplification technique. It is considered as the gold standard for many DNA-based detection methods [32]. Nevertheless, the POC-suitability of this method is often questioned. The expensive equipment, the need for precise temperature protocols and trained staff as well as the operating time were frequently addressed [33–35] (Kim & Easley, 2011). However, many of these arguments can be discussed controversially. Today, low-cost thermal cyclers suitable for POC analyses are available, which generally allow PCR-based rapid and “easy-to-handle” field applications [36, 37]. Further improvement of the PCR components can simplify PCR processing significantly (e.g. by using a lyophilized ready-to-use mastermix) [38]. In addition, it has already been shown that a PCR-LFA combination can produce results in less than 30 min in a POC setting [39]. In our study, the combination of PCR and LFA was selected as a relatively simple, inexpensive, time-saving and POC-compatible detection platform. The multiplex potential of the PCR was used for the implementation of an internal amplification control, which is a unique characteristic of our diagnostic approach. It enables the user to identify PCR inhibition, which was crucial when investigating gill swab samples from deceased fish without extraction of DNA. Without that control even strongly KHV-positive samples would have been tested false-negative due to inhibiting substances in the sample. In addition, the assay described was developed as the basis for an improved analytic approach. The PCR-based detection strategy allows a stronger multiplexing and the developed post-PCR hybridization enables the detection of multiple amplicons (e.g. further infectious agents of interest) through the use of specific hybridization probes.

Overall, our PCR-LFA proved to be a specific, easy-to-use and time-saving POC-compatible test for the detection of KHV-DNA. Regarding gill swab samples, further test series using a higher number of clinical samples should be tested to confirm the number of replicates and sample processing needed to reveal a 100% diagnostic sensitivity.

## Supporting information

S1 Table Samples used for PCR-LFA validation. (DOCX)

S1 Fig Influence of unlabeled helper oligonucleotides for hybridization efficiency—Comprehensive results corresponding to Fig 3. Two helper oligonucleotides, KHV-Helper 1 (H1) and KHV-Helper 2 (H2), were tested under hybridization conditions. Ten pmol helper was added to each hybridization mix including two pmol of the detection probe KHV 109P (rc) BIO. **(A)** KHV-PCR product diluted 1:100. **(B)** KHV-PCR product diluted 1:2000. Tests were carried out in triplicates. [-H]—without helpers; [+H1] − 10 pmol KHV Helper 1; [+H2] − 10 pmol KHV Helper 2; [+H1/2] − 10 pmol KHV Helper 1 + 10 pmol KHV Helper 2. The red arrow indicates the location of the KHV-specific test line. [C] immunoassay control line; [KHV] KHV-specific test line.
(TIFF)

S2 Fig Functionality of the KHV PCR LFA prototype—Comprehensive results corresponding to Fig 4. The assay prototype was tested with the following templates to determine functionality. Lane [1] no PCR control; lane [2] 1x10^8^ KHV copies/μl; lane [3] 1x10^5^ KHV copies/μl, lane [4] 1x10^3^ KHV copies/μl; lane [5] 1x10^2^ KHV copies/μl; lane [6] 1x10^1^ KHV copies/μl; lane [7] no template control; lane [8] forced PCR inhibition a using a polyphenol containing test solution; lane [9] KHV isolate ‘Israel’ (HP 951), unknown concentration; lane [10] KHV isolate ‘Taiwan 832’, unknown concentration. The red arrow indicates the location of the KHV-specific test line. The black arrow indicates the location of the IAC signal. [C]—immunoassay control line; [IAC]—internal amplification control line; [KHV]—KHV-specific test line.
(TIFF)

S3 Fig Raw image used to prepare the left part of Fig 2 (i.e. left of the dotted line). Lateral flow strips were captured by the camera of a mobile phone and were processed using Affinity Designer version 1.8.4 (affinity.serif.com/de/designer/).
(TIFF)

S4 Fig Raw image used to prepare the right part of Fig 2 (i.e. right of the dotted line). Lateral flow strips were captured by the camera of a mobile phone and were processed using Affinity Designer version 1.8.4 (affinity.serif.com/de/designer/).
(TIFF)

S5 Fig Raw image used to prepare Fig 3A showing the influence of unlabeled helper oligonucleotides on hybridization efficiency. Lateral flow strips demonstrate results obtained from experiments using a 1:100 dilution of the KHV-PCR product. Experiments were performed in triplicates. Stripes 1–3: without helpers; stripes 4–6: with 10 pmol KHV-Helper 1; stripes 7–9: with 10 pmol KHV-Helper 2; stripes 10–12: with 10 pmol of both KHV helpers. Stripes marked with an X were not included in Fig 3. S5 Fig is also the raw image of S1A Fig. Lateral flow strips were captured by the camera of a mobile phone and were processed using Affinity Designer version 1.8.4 (affinity.serif.com/de/designer/).
(TIFF)

S6 Fig Raw image used to prepare Fig 3A showing the influence of unlabeled helper oligonucleotides on hybridization efficiency. Lateral flow strips demonstrate results obtained from experiments using a 1:2000 dilution of the KHV-PCR product. Experiments were performed in triplicates. Stripes 1–3: without helpers; stripes 4–6: with 10 pmol KHV-Helper 1; stripes 7–9: with 10 pmol KHV-Helper 2; stripes 10–12: with 10 pmol of both KHV helpers. Stripes marked with an X were not included in Fig 3. S6 Fig is also the raw image of S1B Fig. Lateral flow strips were captured by the camera of a mobile phone and were processed using Affinity Designer version 1.8.4 (affinity.serif.com/de/designer/).
(TIFF)

S7 Fig Raw image used to prepare Fig 4 demonstrating the functionality of the KHV PCR LFA prototype. Fig 4 was prepared using stripes [B -], [B 2], and [B 6]. Stripes marked with an X were not included in Fig 4. S7 Fig is also the raw image of S2 Fig. Lateral flow strips were captured by the camera of a mobile phone and were processed using Affinity Designer version 1.8.4 (affinity.serif.com/de/designer/).
(TIFF)

S8 Fig Raw image of Fig 6: Analytical specificity of the KHV PCR-LFA. The blot stripes were scanned using a Triumph Adler P4035i MFP and the picture was processed by using GIMP 2.10 (https://www.gimp.org). The original labels are as follows: CyHV-1, CyHV-2, CEV, IHNV, SVCV, VHSV, PFRV, Francis—*Francisella noatunensis* subsp. *orientalis*, Alge—*Chlamydomonas reinhardtii*, P.K. I—*Pseudomonas koreensis*, Bac. cer I—*Bacillus cereus*, Aero II—*Aeromonas hydrophila*, St. aur. II—*Staphylococcus aureus*, RB I—redfish, FK I—rainbow trout, Lachs I—salmon, NPC—CyHV-3, NTC—no template control.
(TIFF)
