# Supplementary material for: Diagnostic validation of a rapid and field-applicable PCR-lateral flow test system for point-of-care detection of cyprinid herpesvirus 3 (CyHV-3)
Source: PLoS One. 2020 Oct 30;15(10):e0241420. doi: 10.1371/journal.pone.0241420 (PMC7598509; doi:10.1371/journal.pone.0241420)
Supplement: S1 Table — (DOCX) [file pone.0241420.s001.docx]

**S1 Table. Samples used for PCR-LFA validation.**

| **Sample** | **ID** | **qPCR C_t_-value** | **KVH- copies/µl** | **Interpretation** | **Test validation** |
| --- | --- | --- | --- | --- | --- |
| gill | IA-8 | 18.33 | 7.22x10^6^ | distinct positive | intra-/inter-assay variation |
| gill | UI-44 | 36.56 | 4.56x10^1^ | weakly positive | intra-/inter-assay variation |
| gill | D-22 | 0 | 0 | negative | intra-/inter-assay variation |
| kidney | IA-8 | 23.86 | 1.92x10^5^ | distinct positive | intra-/inter-assay variation |
| kidney | IB-44 | 36.45 | 5.02x10^1^ | weakly positive | intra-/inter-assay variation |
| kidney | D-22 | 0 | 0 | negative | intra-/inter-assay variation |
| gill | D-03 | 0 | 0 | negative | diagnostic sens. & spec. |
| gill | D-06 | 0 | 0 | negative | diagnostic sens. & spec. |
| gill | D-09 | 0 | 0 | negative | diagnostic sens. & spec. |
| gill | D-13 | 0 | 0 | negative | diagnostic sens. & spec. |
| gill | D-14 | 0 | 0 | negative | diagnostic sens. & spec. |
| gill | D-15 | 0 | 0 | negative | diagnostic sens. & spec. |
| gill | D-16 | 0 | 0 | negative | diagnostic sens. & spec. |
| gill | D-18 | 0 | 0 | negative | diagnostic sens. & spec. |
| gill | D-20 | 0 | 0 | negative | diagnostic sens. & spec. |
| gill | D-21 | 0 | 0 | negative | diagnostic sens. & spec. |
| gill | D-23 | 0 | 0 | negative | diagnostic sens. & spec. |
| gill | D-24 | 0 | 0 | negative | diagnostic sens. & spec. |
| gill | D-26 | 0 | 0 | negative | diagnostic sens. & spec. |
| gill | D-29 | 0 | 0 | negative | diagnostic sens. & spec. |
| gill | D-30 | 0 | 0 | negative | diagnostic sens. & spec. |
| gill | D-31 | 0 | 0 | negative | diagnostic sens. & spec. |
| gill | D-32 | 0 | 0 | negative | diagnostic sens. & spec. |
| gill | D-35 | 0 | 0 | negative | diagnostic sens. & spec. |
| gill | D-36 | 0 | 0 | negative | diagnostic sens. & spec. |
| gill | D-37 | 0 | 0 | negative | diagnostic sens. & spec. |
| gill | D-39 | 0 | 0 | negative | diagnostic sens. & spec. |
| gill | IB-9 | 31.33 | 3.35x10^2^ | positive | diagnostic sens. & spec. |
| kidney | UI-15 | 32.56 | 3.60x10^2^ | positive | diagnostic sens. & spec. |
| kidney | UI-25 | 26.53 | 8.21x10^3^ | positive | diagnostic sens. & spec. |
| gill | IB-32 | 33.69 | 1.70x10^2^ | positive | diagnostic sens. & spec. |
| kidney | UI-48 | 31.67 | 1.09x10^3^ | positive | diagnostic sens. & spec. |
| gill | IA-41 | 36.97 | 2.23x10^1^ | weakly positive | diagnostic sens. & spec. |
| kidney | IA-41 | 37.52 | 1.54x10^1^ | weakly positive | diagnostic sens. & spec. |
| gill | IA-43 | 34.22 | 2.16x10^2^ | positive | diagnostic sens. & spec. |
| gill | IA-46 | 32.81 | 3.71x10^2^ | positive | diagnostic sens. & spec. |
| kidney | IB-49 | 33.96 | 1.71x10^2^ | positive | diagnostic sens. & spec. |
| kidney | IB-53 | 31.82 | 7.23x10^2^ | positive | diagnostic sens. & spec. |
| gill | IB-12 | 34.46 | 4.15x10^1^ | positive | diagnostic sens. & spec. |
| gill | IB-14 | 36.42 | 1.13x10^1^ | weakly positive | diagnostic sens. & spec. |
| gill | UI-20 | 27.36 | 1.15x10^4^ | positive | diagnostic sens. & spec. |
| kidney | IA-18 | 32.02 | 9.12x10^2^ | positive | diagnostic sens. & spec. |
| kidney | IA-23 | 30.48 | 2.35x10^3^ | positive | diagnostic sens. & spec. |
| gill | UI-53 | 38.95 | 9.73x10^0^ | weakly positive | diagnostic sens. & spec. |
| gill | IB-41 | 31.50 | 8.99x10^2^ | positive | diagnostic sens. & spec. |
| kidney | IB-46 | 30.32 | 1.99x10^3^ | positive | diagnostic sens. & spec. |
| gill | IB-58 | 35.07 | 8.07x10^1^ | positive | diagnostic sens. & spec. |
| kidney | IB-23 | 37.35 | 6.04x10^0^ | weakly positive | diagnostic sens. & spec. |
| gill | UI-7 | 32.81 | 3.06X10^2^ | positive | diagnostic sens. & spec. |
| gill | UI-24 | 27.43 | 4.51X10^3^ | positive | diagnostic sens. & spec. |
| kidney | IA-18 | 32.02 | 9.12x10^2^ | positive | diagnostic sens. & spec. |
| gill | UI-43 | 34.78 | 9.81x10^1^ | positive | diagnostic sens. & spec. |
| gill | IA-35 | 35.99 | 4.33x10^1^ | positive | diagnostic sens. & spec. |
| gill | IA-49 | 35.90 | 7.19x10^1^ | positive | diagnostic sens. & spec. |
| gill | IA-55 | 35.12 | 1.19x10^2^ | positive | diagnostic sens. & spec. |
| gill | IB-52 | 34.88 | 9.15x10^1^ | positive | diagnostic sens. & spec. |
| kidney | IB-52 | 31.34 | 1.00x10^3^ | positive | diagnostic sens. & spec. |
| gill | IB-20 | 36.11 | 1.38x10^1^ | weakly positive | diagnostic sens. & spec. |
| kidney | IB-20 | 38.24 | 3.33x10^0^ | weakly positive | diagnostic sens. & spec. |
| kidney | UI-9 | 34.24 | 1.19x10^2^ | positive | diagnostic sens. & spec. |
| kidney | UI-17 | 32.34 | 4.18x10^2^ | positive | diagnostic sens. & spec. |
| gill | UI-28 | 32.09 | 2.02x10^2^ | positive | diagnostic sens. & spec. |
| kidney | UI-30 | 37.95 | 4.05x10^0^ | weakly positive | diagnostic sens. & spec. |
| gill | UI-34 | 37.15 | 3.15x10^1^ | weakly positive | diagnostic sens. & spec. |
| gill | IA-38 | 37.81 | 1.26x10^1^ | weakly positive | diagnostic sens. & spec. |
| gill | IA-53 | 35.85 | 4.74X10^1^ | positive | diagnostic sens. & spec. |
| gill | IB-56 | 32.84 | 3.63x10^2^ | positive | diagnostic sens. & spec. |
| gill | IB-10 | 33.78 | 6.52x10^1^ | positive | diagnostic sens. & spec. |
| gill | IB-18 | 31.62 | 2.75x10^2^ | positive | diagnostic sens. & spec. |
| gill | IB-25 | 31.85 | 5.79x10^2^ | positive | diagnostic sens. & spec. |
| kidney | IB-30 | 26.74 | 1.73x10^4^ | positive | diagnostic sens. & spec. |
| kidney | UI-21 | 28.70 | 1.94x10^3^ | positive | diagnostic sens. & spec. |
| kidney | UI-29 | 35.22 | 2.51x10^1^ | positive | diagnostic sens. & spec. |
| gill | IA-13 | 25.51 | 6.52x10^4^ | positive | diagnostic sens. & spec. |
| kidney | IA-15 | 27.85 | 1.40x10^4^ | positive | diagnostic sens. & spec. |
| gill | IA-20 | 18.41 | 5.62x10^6^ | distinct positive | diagnostic sens. & spec. |
| gill | UI-47 | 24.16 | 1.38x10^5^ | positive | diagnostic sens. & spec. |
| kidney | IA-52 | 25.82 | 4.17x10^4^ | positive | diagnostic sens. & spec. |
| kidney | IB-40 | 28.64 | 6.20x10^3^ | positive | diagnostic sens. & spec. |
| gill | IB-48 | 35.64 | 5.49x10^1^ | positive | diagnostic sens. & spec. |
| gill | IB-54 | 33.20 | 2.85x10^2^ | positive | diagnostic sens. & spec. |
| kidney | IB-54 | 32.12 | 5.92x10^2^ | positive | diagnostic sens. & spec. |
| gill | UI-55 | 38.94 | 5.88x10^0^ | weakly positive | diagnostic sens. & spec. |
| kidney | UI-55 | 38.78 | 6.55x10^0^ | weakly positive | diagnostic sens. & spec. |
| kidney | D-02 | 0 | 0 | negative | diagnostic sens. & spec. |
| kidney | D-06 | 0 | 0 | negative | diagnostic sens. & spec. |
| kidney | D-07 | 0 | 0 | negative | diagnostic sens. & spec. |
| kidney | D-09 | 0 | 0 | negative | diagnostic sens. & spec. |
| kidney | D-10 | 0 | 0 | negative | diagnostic sens. & spec. |
| kidney | D-13 | 0 | 0 | negative | diagnostic sens. & spec. |
| kidney | D-14 | 0 | 0 | negative | diagnostic sens. & spec. |
| kidney | D-15 | 0 | 0 | negative | diagnostic sens. & spec. |
| kidney | D-16 | 0 | 0 | negative | diagnostic sens. & spec. |
| kidney | D-18 | 0 | 0 | negative | diagnostic sens. & spec. |
| kidney | D-20 | 0 | 0 | negative | diagnostic sens. & spec. |
| kidney | D-21 | 0 | 0 | negative | diagnostic sens. & spec. |
| kidney | D-24 | 0 | 0 | negative | diagnostic sens. & spec. |
| kidney | D-26 | 0 | 0 | negative | diagnostic sens. & spec. |
| kidney | D-29 | 0 | 0 | negative | diagnostic sens. & spec. |
| kidney | D-30 | 0 | 0 | negative | diagnostic sens. & spec. |
| kidney | D-31 | 0 | 0 | negative | diagnostic sens. & spec. |
| kidney | D-32 | 0 | 0 | negative | diagnostic sens. & spec. |
| kidney | D-35 | 0 | 0 | negative | diagnostic sens. & spec. |
| kidney | D-36 | 0 | 0 | negative | diagnostic sens. & spec. |
| kidney | D-37 | 0 | 0 | negative | diagnostic sens. & spec. |
| kidney | D-39 | 0 | 0 | negative | diagnostic sens. & spec. |
| gill swab | 1 | 22.89 | 4.75x10^4^ | distinct positive | diagnostic sens. & spec. |
| gill swab | 2 | 23.61 | 2.87x10^4^ | distinct positive | diagnostic sens. & spec. |
| gill swab | 3 | 23.38 | 3.35x10^4^ | distinct positive | diagnostic sens. & spec. |
| gill swab | 4 | 17.99 | 1.51x10^6^ | distinct positive | diagnostic sens. & spec. |
| gill swab | 5 | 17.40 | 1.45x10^7^ | distinct positive | diagnostic sens. & spec. |
| gill swab | 6 | 19.55 | 5.01x10^5^ | distinct positive | diagnostic sens. & spec. |
| gill swab | 7 | 23.64 | 2.80x10^4^ | distinct positive | diagnostic sens. & spec. |
| gill swab | 8 | 20.76 | 2.14x10^5^ | distinct positive | diagnostic sens. & spec. |
| gill swab | 9 | 20.50 | 1.87x10^6^ | distinct positive | diagnostic sens. & spec. |
| gill swab | 10 | 25.32 | 7.66x10^4^ | positive | diagnostic sens. & spec. |
| gill swab | 11 | 21.37 | 1.05x10^6^ | distinct positive | diagnostic sens. & spec. |
| gill swab | 12 | 26.30 | 4.27x10^3^ | positive | diagnostic sens. & spec. |
| gill swab | 13 | 24.76 | 1.27x10^4^ | positive | diagnostic sens. & spec. |
| gill swab | 14 | 18.91 | 7.88x10^5^ | distinct positive | diagnostic sens. & spec. |
| gill swab | 15 | 28.87 | 6.99x10^2^ | positive | diagnostic sens. & spec. |
| gill swab | 16 | 30.01 | 3.12x10^2^ | positive | diagnostic sens. & spec. |
| gill swab | 17 | 22.02 | 8.81x10^4^ | distinct positive | diagnostic sens. & spec. |
| gill swab | 18 | 26.09 | 4.95x10^3^ | positive | diagnostic sens. & spec. |
| gill swab | 19 | 29.00 | 6.36x10^2^ | positive | diagnostic sens. & spec. |
| gill swab | 20 | 20.62 | 2.36x10^5^ | distinct positive | diagnostic sens. & spec. |
