# Supplementary figures and images for: Diagnostic validation of a rapid and field-applicable PCR-lateral flow test system for point-of-care detection of cyprinid herpesvirus 3 (CyHV-3)
Source: PLoS One. 2020 Oct 30;15(10):e0241420. doi: 10.1371/journal.pone.0241420 (PMC7598509; doi:10.1371/journal.pone.0241420)

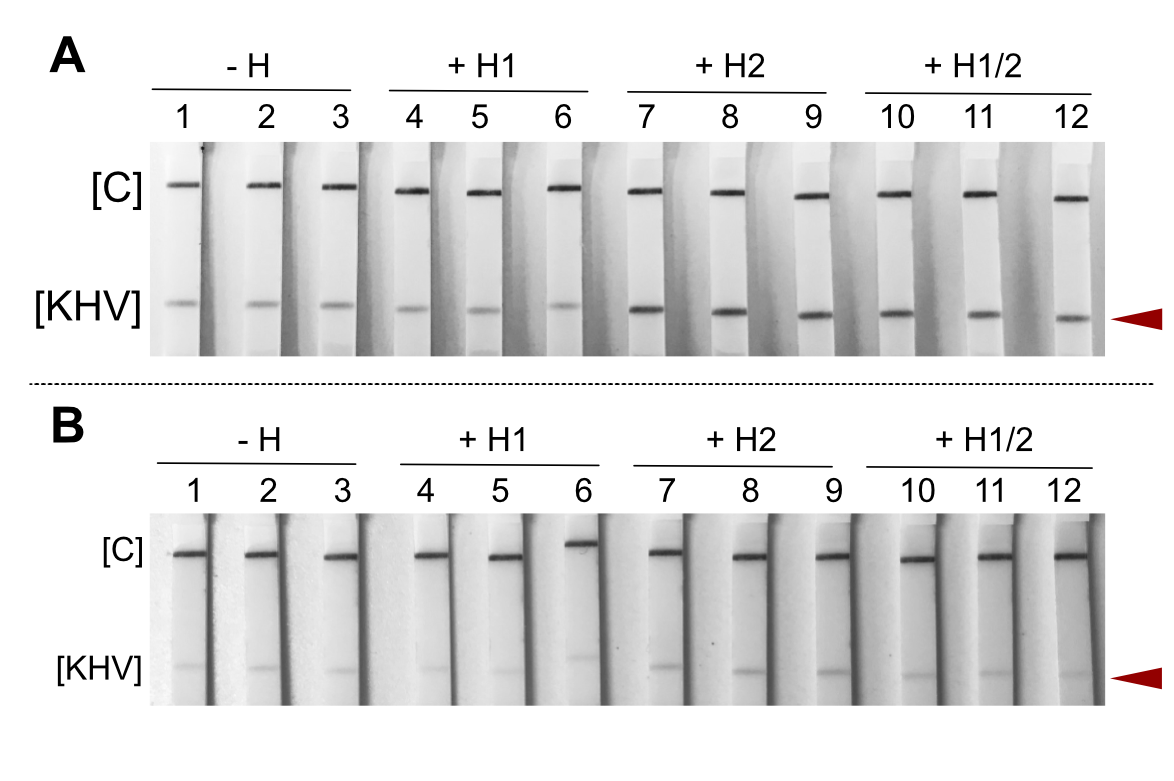

Supplement: S1 Fig — Two helper oligonucleotides, KHV-Helper 1 (H1) and KHV-Helper 2 (H2), were tested under hybridization conditions. Ten pmol helper was added to each hybridization mix including two pmol of the detection probe KHV 109P (rc) BIO. (A) KHV-PCR product diluted 1:100. (B) KHV-PCR product diluted 1:2000. Tests were carried out in triplicates. [-H]—without helpers; [+H1] − 10 pmol KHV Helper 1; [+H2] − 10 pmol KHV Helper 2; [+H1/2] − 10 pmol KHV Helper 1 + 10 pmol KHV Helper 2. The red arrow indicates the location of the KHV-specific test line. [C] immunoassay control line; [KHV] KHV-specific test line. (TIFF) [file pone.0241420.s002.tiff]

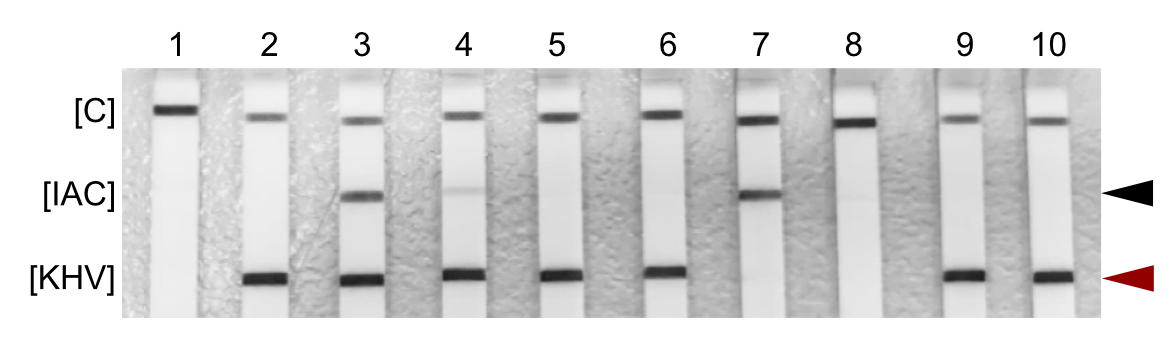

Supplement: S2 Fig — The assay prototype was tested with the following templates to determine functionality. Lane [1] no PCR control; lane [2] 1x10^8^ KHV copies/μl; lane [3] 1x10^5^ KHV copies/μl, lane [4] 1x10^3^ KHV copies/μl; lane [5] 1x10^2^ KHV copies/μl; lane [6] 1x101 KHV copies/μl; lane [7] no template control; lane [8] forced PCR inhibition a using a polyphenol containing test solution; lane [9] KHV isolate ‘Israel’ (HP 951), unknown concentration; lane [10] KHV isolate ‘Taiwan 832’, unknown concentration. The red arrow indicates the location of the KHV-specific test line. The black arrow indicates the location of the IAC signal. [C]—immunoassay control line; [IAC]—internal amplification control line; [KHV]—KHV-specific test line. (TIFF) [file pone.0241420.s003.tiff]

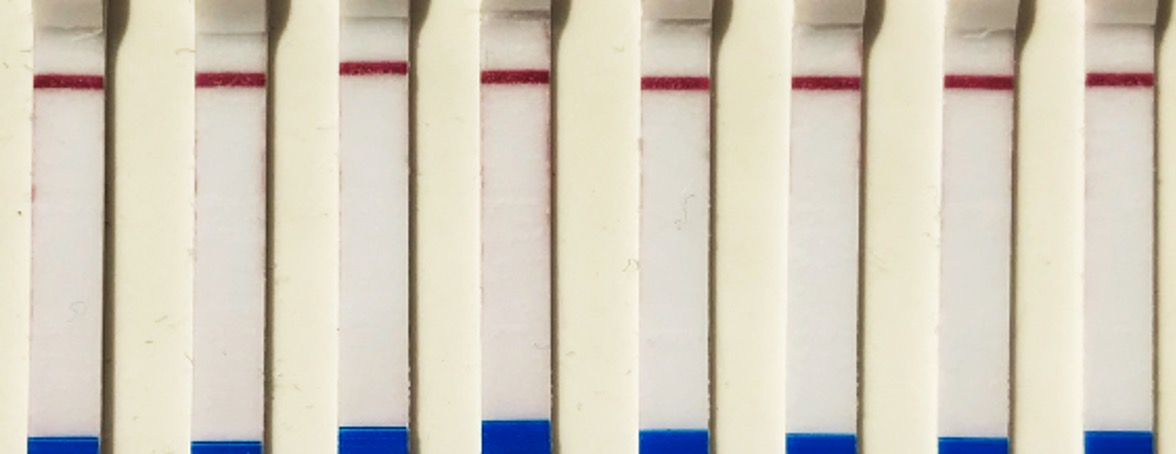

Supplement: S3 Fig — Lateral flow strips were captured by the camera of a mobile phone and were processed using Affinity Designer version 1.8.4 (affinity.serif.com/de/designer/). (TIFF) [file pone.0241420.s004.tiff]

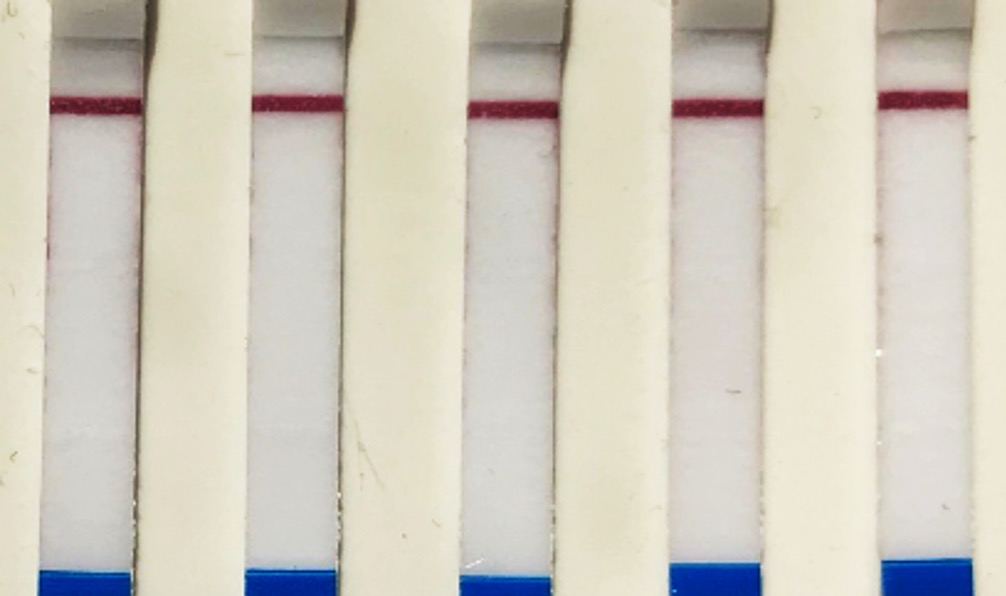

Supplement: S4 Fig — Lateral flow strips were captured by the camera of a mobile phone and were processed using Affinity Designer version 1.8.4 (affinity.serif.com/de/designer/). (TIFF) [file pone.0241420.s005.tiff]

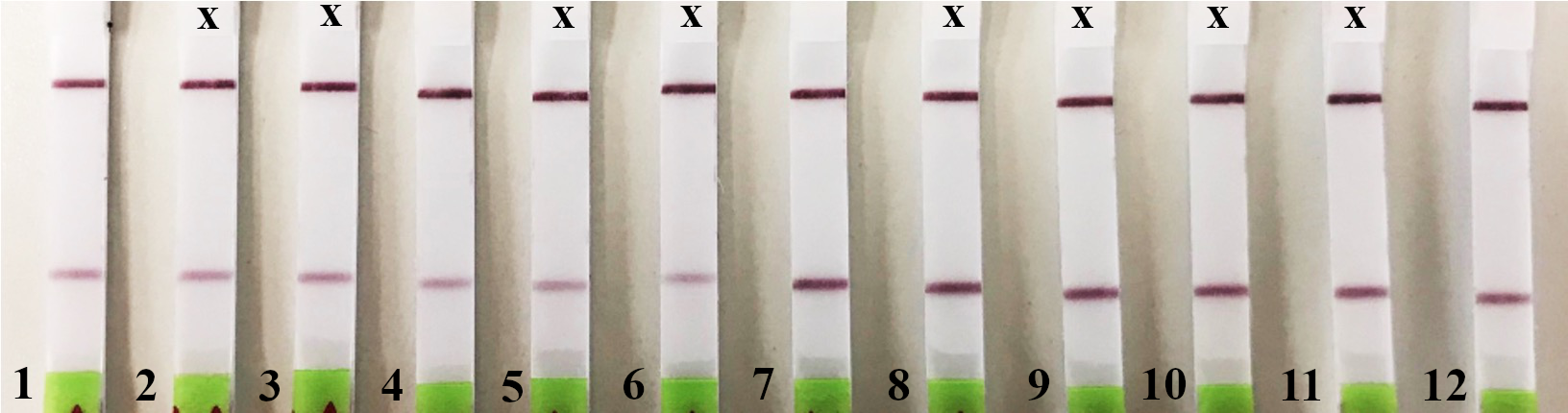

Supplement: S5 Fig — Lateral flow strips demonstrate results obtained from experiments using a 1:100 dilution of the KHV-PCR product. Experiments were performed in triplicates. Stripes 1–3: without helpers; stripes 4–6: with 10 pmol KHV-Helper 1; stripes 7–9: with 10 pmol KHV-Helper 2; stripes 10–12: with 10 pmol of both KHV helpers. Stripes marked with an X were not included in Fig 3. S5 Fig is also the raw image of S1A Fig. Lateral flow strips were captured by the camera of a mobile phone and were processed using Affinity Designer version 1.8.4 (affinity.serif.com/de/designer/). (TIFF) [file pone.0241420.s006.tiff]

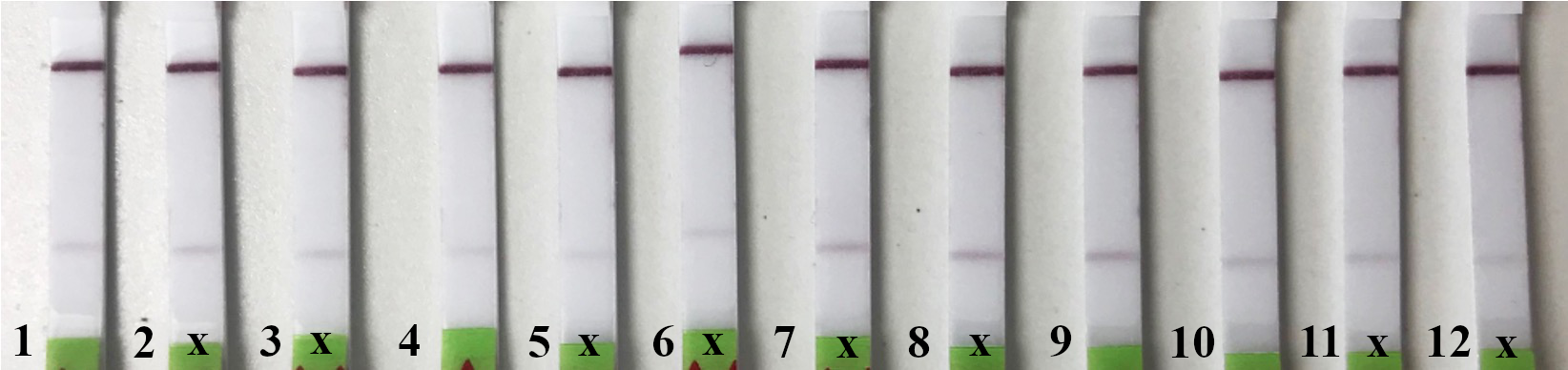

Supplement: S6 Fig — Lateral flow strips demonstrate results obtained from experiments using a 1:2000 dilution of the KHV-PCR product. Experiments were performed in triplicates. Stripes 1–3: without helpers; stripes 4–6: with 10 pmol KHV-Helper 1; stripes 7–9: with 10 pmol KHV-Helper 2; stripes 10–12: with 10 pmol of both KHV helpers. Stripes marked with an X were not included in Fig 3. S6 Fig is also the raw image of S1B Fig. Lateral flow strips were captured by the camera of a mobile phone and were processed using Affinity Designer version 1.8.4 (affinity.serif.com/de/designer/). (TIFF) [file pone.0241420.s007.tiff]

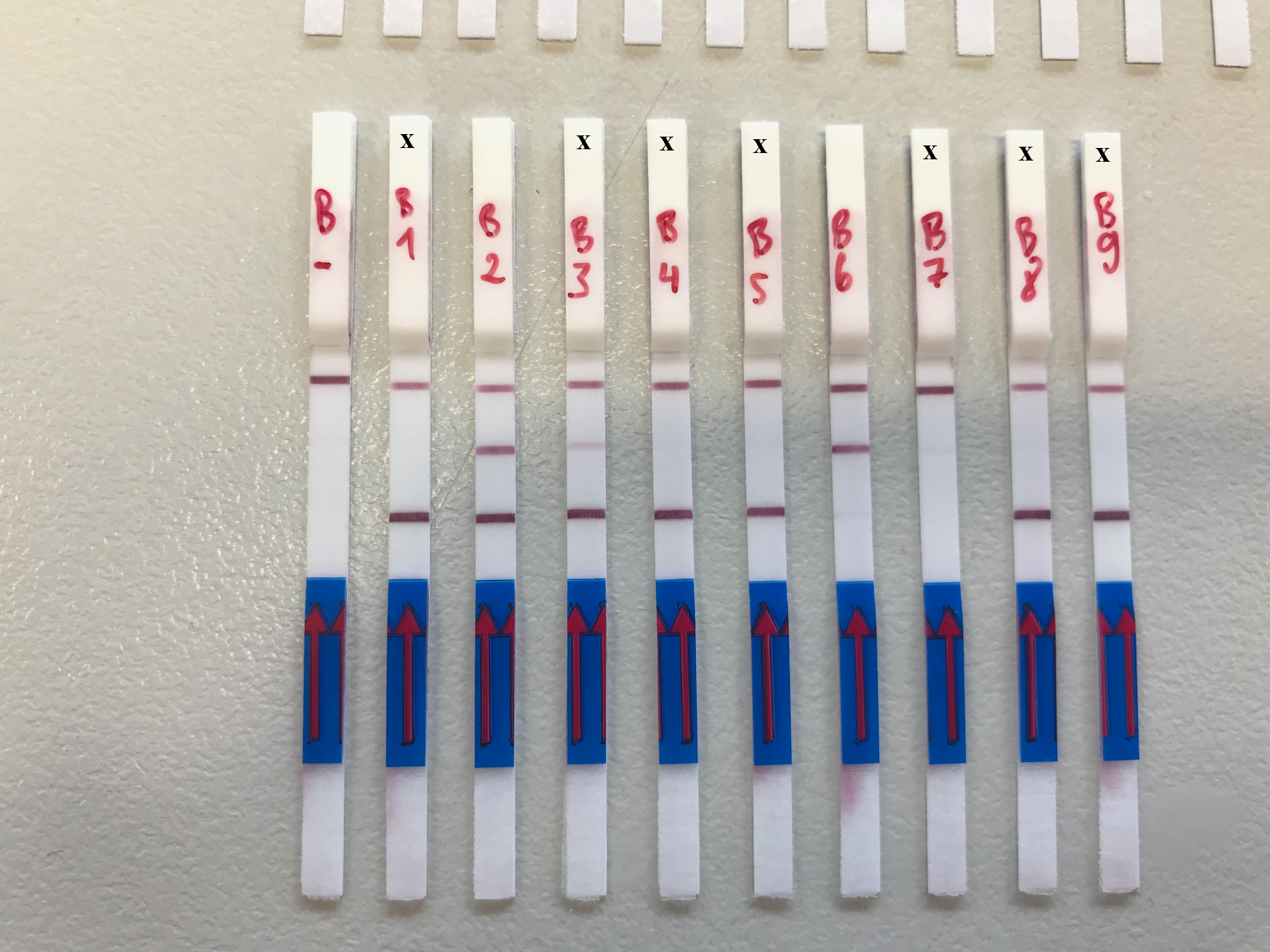

Supplement: S7 Fig — Fig 4 was prepared using stripes [B -], [B 2], and [B 6]. Stripes marked with an X were not included in Fig 4. S7 Fig is also the raw image of S2 Fig. Lateral flow strips were captured by the camera of a mobile phone and were processed using Affinity Designer version 1.8.4 (affinity.serif.com/de/designer/). (TIFF) [file pone.0241420.s008.tiff]

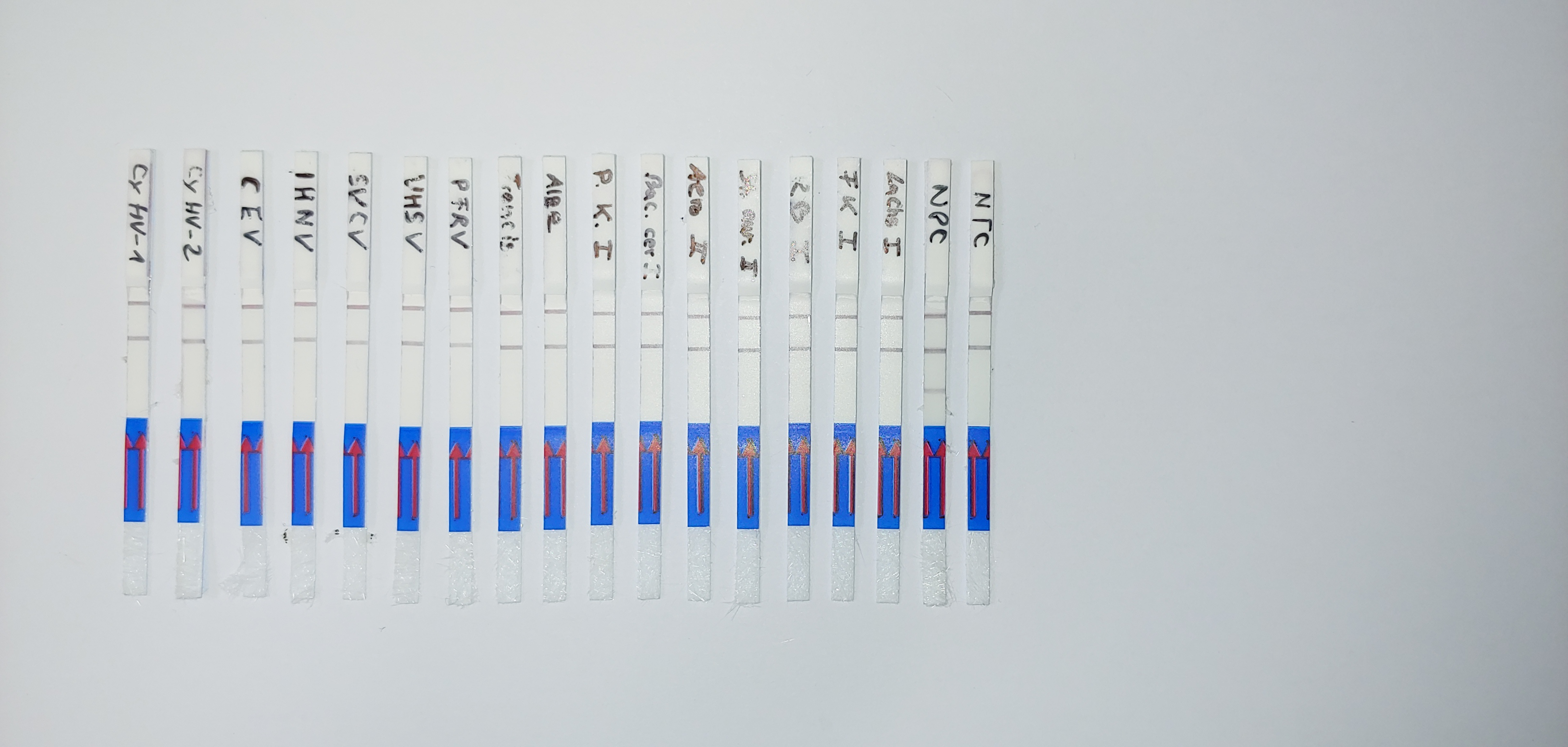

Supplement: S8 Fig — The blot stripes were scanned using a Triumph Adler P4035i MFP and the picture was processed by using GIMP 2.10 (https://www.gimp.org). The original labels are as follows: CyHV-1, CyHV-2, CEV, IHNV, SVCV, VHSV, PFRV, Francis—Francisella noatunensis subsp. orientalis, Alge—Chlamydomonas reinhardtii, P.K. I—Pseudomonas koreensis, Bac. cer I—Bacillus cereus, Aero II—Aeromonas hydrophila, St. aur. II—Staphylococcus aureus, RB I—redfish, FK I—rainbow trout, Lachs I—salmon, NPC—CyHV-3, NTC—no template control. (TIFF) [file pone.0241420.s009.tiff]
